# Patients with progranulin mutations overlap with the progressive dysexecutive syndrome: towards the definition of a frontoparietal dementia phenotype

**DOI:** 10.1093/braincomms/fcaa126

**Published:** 2020-11-05

**Authors:** Miguel Tábuas-Pereira, Maria Rosário Almeida, Diana Duro, Marisa Lima, João Durães, Rita Guerreiro, José Brás, Inês Baldeiras, Isabel Santana

**Affiliations:** Department of Neurology, Centro Hospitalar e Universitário de Coimbra, Coimbra 3000-045, Portugal; Faculty of Medicine, University of Coimbra, Coimbra 3000-370, Portugal; Center for Innovative Biomedicine and Biotechnology, University of Coimbra, Coimbra 3004-517, Portugal; Centro Académico Clínico de Coimbra, University of Coimbra, Coimbra 3004-517, Portugal; Center for Innovative Biomedicine and Biotechnology, University of Coimbra, Coimbra 3004-517, Portugal; Centro Académico Clínico de Coimbra, University of Coimbra, Coimbra 3004-517, Portugal; Department of Neurology, Centro Hospitalar e Universitário de Coimbra, Coimbra 3000-045, Portugal; Faculty of Medicine, University of Coimbra, Coimbra 3000-370, Portugal; University of Coimbra, Center for Research in Neuropsychology and Cognitive Behavioral Intervention (CINEICC), Faculty of Psychology and Educational Sciences, Coimbra 3000-115, Portugal; Department of Neurology, Centro Hospitalar e Universitário de Coimbra, Coimbra 3000-045, Portugal; Centro Académico Clínico de Coimbra, University of Coimbra, Coimbra 3004-517, Portugal; University of Coimbra, Center for Research in Neuropsychology and Cognitive Behavioral Intervention (CINEICC), Faculty of Psychology and Educational Sciences, Coimbra 3000-115, Portugal; Department of Neurology, Centro Hospitalar e Universitário de Coimbra, Coimbra 3000-045, Portugal; Center for Neurodegenerative Science, Van Andel Institute, Grand Rapids, MI 49503, USA; Division of Psychiatry and Behavioral Medicine, Michigan State University College of Human Medicine, Grand Rapids, MI 49503, USA; Center for Neurodegenerative Science, Van Andel Institute, Grand Rapids, MI 49503, USA; Division of Psychiatry and Behavioral Medicine, Michigan State University College of Human Medicine, Grand Rapids, MI 49503, USA; Department of Neurology, Centro Hospitalar e Universitário de Coimbra, Coimbra 3000-045, Portugal; Faculty of Medicine, University of Coimbra, Coimbra 3000-370, Portugal; Center for Innovative Biomedicine and Biotechnology, University of Coimbra, Coimbra 3004-517, Portugal; Centro Académico Clínico de Coimbra, University of Coimbra, Coimbra 3004-517, Portugal; Department of Neurology, Centro Hospitalar e Universitário de Coimbra, Coimbra 3000-045, Portugal; Faculty of Medicine, University of Coimbra, Coimbra 3000-370, Portugal; Center for Innovative Biomedicine and Biotechnology, University of Coimbra, Coimbra 3004-517, Portugal; Centro Académico Clínico de Coimbra, University of Coimbra, Coimbra 3004-517, Portugal

We read with great interest the article by Townley *et al.* (2020), which described a series of 55 patients with vprogressive early-onset dementia with a predominant impairment in the executive functions with probable Alzheimer’s disease. They thoroughly characterize these patients, who show a consistent impairment of executive functions rather than behavioural changes, defining the progressive dysexecutive syndrome. These patients consistently revealed hypometabolism in parietofrontal brain regions. All the patients had positive amyloid biomarkers and the great majority (48/55) also had positive tau biomarkers. Two of the patients had pathological confirmation of the diagnosis. Yet, genetic test was only performed in eight patients and only looking for the autosomal dominant mutations associated with Alzheimer’s disease. In fact, the authors carefully state that this presentation may not be specific to Alzheimer’s disease, but report only those cases, as Alzheimer’s disease is probably the most common cause of this syndrome. This is a very interesting paper, defining a new subset of patients with cognitive impairment. Deep phenotyping is a potent tool for personalized medicine and for the understanding of new syndromic entities.

In this letter, we describe our cohort of patients with *GRN* (progranulin) mutation, including Cerebrospinal Fluid (CSF) findings, and discuss the similarities with those of the proposed progressive dysexecutive syndrome.

The serum GRN level assessments and the mutation analysis in this gene were performed as previously described by our group (Almeida *et al.*, 2014). CSF samples were collected as part of the routine clinical diagnostic protocol. Pre-analytical and analytical procedures were done in accordance with the Alzheimer’s Association guidelines for CSF biomarker determination (Mattsson *et al.*, 2011). CSF samples were collected in sterile polypropylene tubes, immediately centrifuged at 1800 × *g* for 10 min at 4°C, aliquoted into polypropylene tubes and stored at −80°C until analysis. CSF Aβ42, total-Tau and phosporylated-Tau (p-Tau) were measured separately by commercially available sandwich ELISA kits (Innotest, Fujirebio, Belgium), as previously described (Kapaki *et al.*, 2001; Baldeiras *et al.*, 2009). External quality control of the assays was performed under the scope of the Alzheimer’s Association Quality Control Program for CSF Biomarkers (Mattsson *et al.*, 2011). Neuropsychological studies’ methodology and results are reported elsewhere (Lima *et al.*, 2020). The study was approved by the ethics committee of our hospital, and biological samples were obtained following written informed consent from the legal representatives.

Interestingly, it has been previously demonstrated that *GRN* mutation carriers may show a phenotype resembling Alzheimer’s disease, with marked memory impairment associated with frontal lobe changes (Le Ber *et al.*, 2008; Kelley *et al.*, 2009; Hallam *et al.*, 2014). Peculiar features like early visuospatial and working memory deficits (Hallam *et al.*, 2014) and parietal signs such as apraxia and dyscalculia have also been described (Rohrer *et al.*, 2008), which correlate with early temporal, parietal and insular atrophy (Whitwell *et al.*, 2007; Rohrer *et al.*, 2015). There are additional similarities between *GRN* patients and the group reported in the paper by Townley *et al.* (2020). Of notice, two patients had parkinsonism, with one of them having a previous diagnosis of corticobasal syndrome. Twenty-four out of 31 patients had language involvement, and 28 out of 40 had ideomotor apraxia. All of these findings are common on *GRN* mutation carriers (Le Ber *et al.*, 2008; Hallam *et al.*, 2014). In our cohort, *GRN* mutation carriers have worse scores in executive functions, initiative and psychomotor control than both Alzheimer’s disease and behavioural-variant frontotemporal dementia (bvFTD) patients matched for age, severity and education (Lima *et al.*, 2020). Regarding memory, *GRN* patients presented with a transitional performance between sporadic bvFTD and Alzheimer’s disease. They are better than Alzheimer’s disease on measures of both immediate and delayed recall but worse than sporadic bvFTD. Focusing on parietal functions, these patients also exhibit a visuospatial pattern of performance that includes features of both sporadic bvFTD and Alzheimer’s disease patients, with lack of elements and gestalt changes (features from Alzheimer’s disease) and also with perseveration and lack of planning (sporadic bvFTD features). Consistent with the progressive dysexeutive syndrome patients, *GRN* mutation carriers have been shown to have more complaints on executive functions and less complaints on social changes than other ubiquitin-positive Frontotemporal dementia (FTD) (Van Deerlin *et al.*, 2007). In fact, this different profile of *GRN* mutation carriers has been previously reported (Beck *et al.*, 2008), with these patients characteristically less frequently showing disinhibition, loss of empathy, aggression and obsessive behaviour.

Regarding Alzheimer’s disease biomarkers, of our cohort of *GRN* mutation carriers, 21 patients have had collected CSF Alzheimer’s disease biomarkers. Of these, six (28.6%) had abnormal CSF amyloid-β_42_, with an additional four (adding up to 47.6%) having very borderline values. In terms of CSF total-Tau, 16 (76.2%) had increased values. Concerning p-Tau, eight (38.1%) had increased values. Considering the A/T/N classification scheme (Jack *et al.*, 2016), eight (38.1%) patients are A−/T+/N+ and another three (14.3%) are A+/T−/N+. Together with the neuropsychological data, this might have led to a diagnosis of Alzheimer’s disease or Alzheimer’s disease variants in over half of the patients by some criteria (Dubois *et al.*, 2014). However, no patients were A+/T+/N+ or A+/T+/N− (two A+/T−/N+ had borderline p-Tau and one A−/T+/N+ had borderline CSF amyloid-β_42_).

The neuropsychological and imaging data place *GRN* mutation carriers in an intermediate position between Alzheimer’s disease and FTD. For a number of reasons, they stand out of the standard FTD phenotype: they frequently have parietal and memory impairment; they show insular and parietal atrophy; they may present as corticobasal syndrome; and they are very rarely associated with amyotrophic lateral sclerosis (Guerreiro *et al.*, 2020). The mixed CSF biomarkers profile further complicate the distinction.

This report has some limitations, the A/T/N comprises other biomarkers that we did not include in this report and could give additional information. The use of both total-Tau and p-Tau may have some limitations, given their correlation. However, this report gives data on real-world evidence with a substantial amount of patients, whose results may guide both further research studies and the clinical approach.

In conclusion, *GRN*-associated FTD has a great overlap with the progressive dysexecutive syndrome patients. Hence, this new subset of patients defined by Townley *et al.* (2020) may be very much justified, as it may harbour not only a specific anatomical pattern of parietofrontal dysfunction but also a specific genetic background, such as *GRN*, and possibly others, such as the recently reported *CYLD* (Dobson-Stone *et al.*, 2020; Tábuas-Pereira *et al.*, 2020). *GRN* mutations should always be suspected in this setting, regardless of CSF biomarkers, especially if new gene-targeting treatments arise.

## Data availability

Data are shown within the paper. Additional information is available upon request to the corresponding author.

## Competing interests

The authors have no competing interests to declare.
